# Numeracy in Adolescents With Type 1 Diabetes: Assessment and Application of a Video Game Intervention

**DOI:** 10.7759/cureus.108251

**Published:** 2026-05-04

**Authors:** Ereny Bassilious, Devin Johnson, Elif Bilgic, Adam Dubrowski, Alan Schwartz, Kahono Hirasawa, Farid Mahmud

**Affiliations:** 1 Pediatric Endocrinology, McMaster University, Hamilton, CAN; 2 McMaster Health Education Research, Innovation &amp; Theory (MERIT) Centre, McMaster University, Hamilton, CAN; 3 Pediatrics, McMaster University, Hamilton, CAN; 4 maxSIMhealth Group, Ontario Tech University, Oshawa, CAN; 5 Medical Education, University of Illinois at Chicago, Chicago, USA; 6 Health Sciences, McMaster University, Hamilton, CAN; 7 Pediatric Endocrinology, University of Toronto, Toronto, CAN

**Keywords:** numeracy, paediatric endocrinology, simulation in medical education, type 1 diabetes, video game intervention

## Abstract

Background: Numeracy is about understanding and using numbers daily, and low numeracy leads to poor clinical outcomes in adults with diabetes. However, the pediatric literature on this topic is limited.

Objectives: Hence, through this pilot project, we are exploring the role of a novel video game intervention to enhance diabetes-related numeracy and related measures.

Research design and methods: From May 2011 to July 2011, using data from focus groups with diabetes team members and adolescents, we created a video game addressing diabetes numeracy. Diabetes numeracy was assessed using the Adolescent Diabetes Numeracy Test (aDNT14). Correlation of aDNT14 was calculated with cumulative A1C, quality-of-life (QL-PedsQL 3.0 inventory), and demographic data. General numeracy, assessed using the Wide Range Achievement Test, Fourth Edition (WRAT-4), and health literacy, assessed using the Rapid Estimate of Adolescent Literacy in Medicine-Teen (REALM-Teen), were also assessed. Participants were 13-18 years old with type 1 diabetes who had high proficiency in the English language and had no known learning disability, psychiatric/behavioral diagnosis, or visual or hearing impairment. Participants played the video game in three sessions, and their diabetes numeracy was re-evaluated. For retention of gains from the video game, baseline measures were re-evaluated within one month following the last session and cumulative A1C over six months. Analysis included descriptive and inferential statistics.

Results: Forty-two adolescents participated. Baseline A1C and numeracy scores were significantly associated. Diabetes numeracy was also significantly related to scores of general numeracy and literacy. However, the video game intervention had no long-term impact on A1C, diabetes-related numeracy, or QL measures.

Conclusion: Although our diabetes-focused video game intervention dedicated to literacy and numeracy had no long-term impact on glycemic control, diabetes-focused numeracy, or QL measures, our findings support the feasibility and potential value of an experiential, game-based approach for improving type 1 diabetes. Hence, there is a need for larger studies with flexible and longer exposure and/or integration into routine education to explore sustained clinical and patient-reported impacts.

## Introduction

Type 1 diabetes is a common chronic disease in children and adolescents [1]. Patients with type 1 diabetes engage in frequent, daily diabetes related calculations. Numeracy, which is about understanding and applying numbers to daily life, directly affects their ability to self-manage their diabetes [2]. For example, effective self-management requires frequent, on-the-spot use of numbers (e.g., carbohydrate estimation, insulin dose calculation, and interpretation of blood glucose (BG) values), making numeracy a central component of daily care. Furthermore, low numeracy is prevalent (55% of Canadians have low numeracy skills), and low numeracy has been implicated in poorer management of multiple chronic health conditions such as asthma and obesity [3]. In adults with diabetes, both low health literacy and low numeracy were associated with poor clinical outcomes [4]. However, these associations may not translate directly to younger populations. Adolescents are still acquiring numeracy skills, often share diabetes-management tasks with caregivers, and experience developmental and contextual factors (e.g., competing social priorities, transitioning expectations for self-management, and heavy reliance on technology supports) that can modify how numeracy affects outcomes. Additionally, the current generation of children and adolescents is exposed to very different teaching methodologies, which are heavily influenced by technology [4,5]. Also, in our setting, diabetes care for children and adolescents is typically delivered in structured, specialized clinics with equivalent access, which may mitigate access-related challenges that are prominent in some adult studies [5].

Adolescents also represent a population at special risk [6] for frequent deterioration of BG control, partly due to growth and puberty, psychosocial variables, increasing complexity of self-management due to the intense treatment regimens, and a mismatch between the health team’s expectations and the capabilities of the adolescent patient [7]. Quality of life (QL), as a patient-reported outcome, is plausibly influenced by the cognitive and emotional load of type 1 diabetes management, while recognizing that QL is multifaceted and might overlap with other psychosocial constructs (e.g., illness perceptions and beliefs) [8]. Interestingly, self-reported diabetes-related QL is not associated with measures of glycemic control, leaving a question as to whether other factors, such as comfort with health literacy or numeracy skills, can be associated with the QL of patients with diabetes [7].

Finally, the current generation of adolescents embraces technology and often prefers developmentally appropriate, experiential learning approaches [9]. Previously described technology interventions aimed at improving numeracy have only been applied to adults. These interventions have been relatively simple and “low tech” (e.g., in-clinic goal-setting, structured counseling, text message reminders, simple web-based feedback portals, and self-directed computer modules), and most have failed to show a sustained effect after the intervention was discontinued [10]. As well, most of these interventions relied on factors external to the patient, using reminders and counseling from healthcare team members. This contrasts with the use of gaming technology that is patient-centered and patient-driven. Video games in particular are an excellent medium for experiential learning as they provide the participant with concrete experiences and opportunities for active experimentation [11, 12]. Specifically, video games offer an alternative medium that can embed repeated practice of diabetes numeracy decisions in an engaging, simulated environment aligned with how adolescents learn and spend leisure time. Video game technology has also been successfully implemented in health professional education [13, 14]. However, there have been limited studies to date exploring the role of developmentally appropriate technology, such as video games, for improving numeracy skills in adolescents.

Problem statement

The relationship between numeracy and glycemic control in adolescents had not been fully explored. Mulvaney et al. demonstrated an association between numeracy and glycemic control (A1C) in a relatively ethnically homogenous group (90% were white) of adolescents with type 1 diabetes [15]. However, data are lacking on the relationship between numeracy and glycemic control, as well as the relationship between numeracy and QL in ethnically diverse adolescents. Furthermore, there are limited studies examining the effect of developmentally appropriate, interactive, and adolescent-focused educational interventions to improve diabetes-specific numeracy skills. Hence, the purpose of our pilot study is to explore the impact of an innovative video game intervention to improve type 1 diabetes-specific numeracy skills and glycemic control.

## Materials and methods

**Figure 1:**
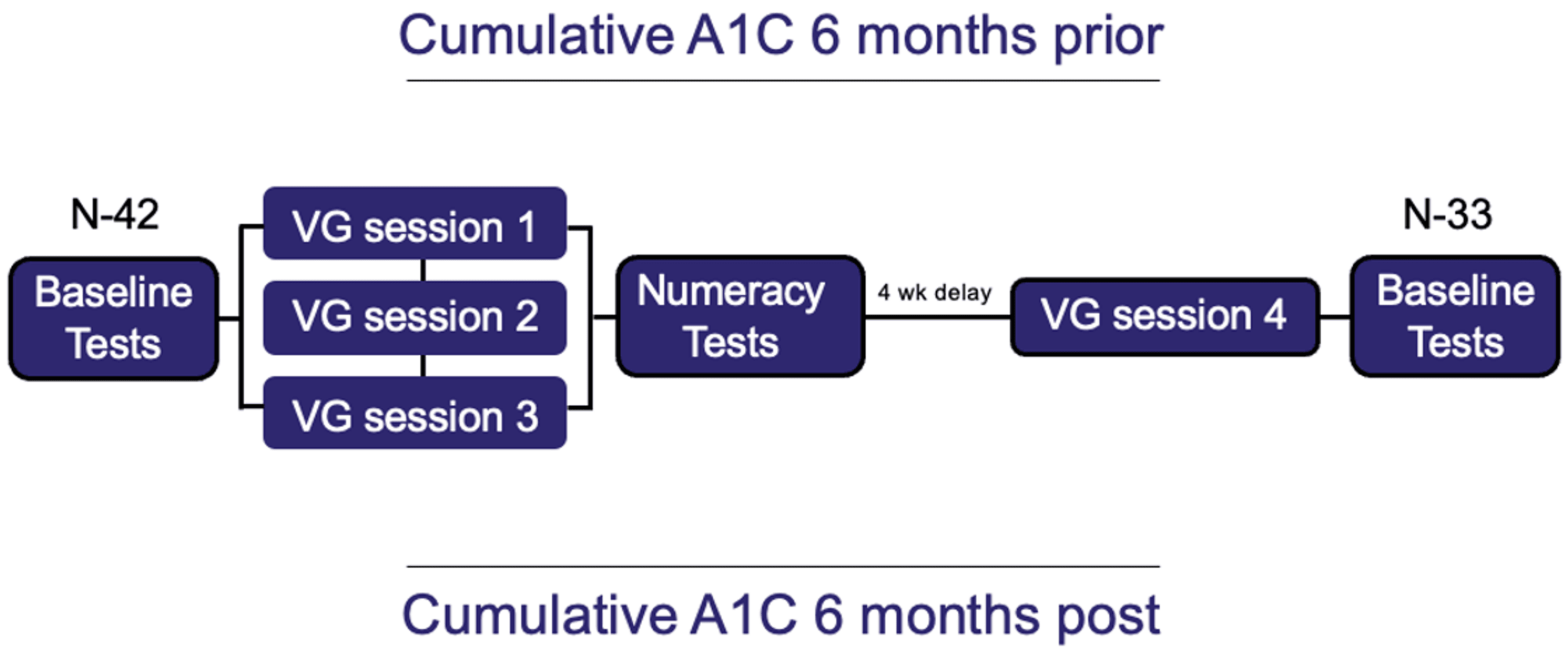
Study design A1C: hemoglobin A1C; VG: video game

We report legacy data from a pilot, quasi-experimental research study that followed a pre-test/post-test design to measure differences in diabetes-related numeracy, general numeracy, literacy, QL, and glycemic control for adolescent patients with type 1 diabetes (Figure 1).

Specific objectives

The primary objective of this study is to apply an interactive video game intervention aimed at improving diabetes-related numeracy and examining its effects on the outcome measures of diabetes related numeracy, glycemic control (A1C), and QL. The secondary objective is to assess the association between baseline diabetes-related numeracy with glycemic control and QL in adolescents with type 1 diabetes.

Intervention: data gathering for game content and design

We conducted meetings with an advisory group composed of diabetes team members at our institution, including diabetes nurse educators, social workers, physicians, dietitians, and child life specialists, to determine the most essential domains of diabetes numeracy to address in the video game design. In these meetings, to prioritize diabetes numeracy content for inclusion in the game, we used a structured Q-sort-style consensus process [16]. We presented the advisory group members with 12 predetermined diabetes numeracy skills (chosen through consensus by the authors and experts in diabetes), who independently ranked the topics they felt were most important to address in the game through a preselected number of Post-it notes. The 12 skills are as follows: Check BG four times per day (QID) and administer insulin with appropriate technique and on time, check ketones, interpret BG results, adjust dose according to BG reading, interpret ketones, draw appropriate type and amount of insulin, count carbohydrates, manage intercurrent illness, calculate correct insulin sensitivity factor (ISF), calculate dose of insulin using insulin to carbohydrate ratio, and judge when to seek medical attention. As a group, these choices were further discussed until consensus on the top five most important topics in diabetes numeracy was reached for integration into the video game. These five essential diabetes numeracy topics identified included (1) carbohydrate counting, (2) insulin dose calculation per meal, (3) BG interpretation, (4) correction insulin calculation, and (5) exercise and BG. Using these data and in collaboration with education scientists and technology developers, we created a video game de novo addressing diabetes-related numeracy, whereby instructions related to the five essential diabetes management skills were embedded. We also had adolescent input to enhance interactivity and stimulation by adding features.

**Figure 2:**
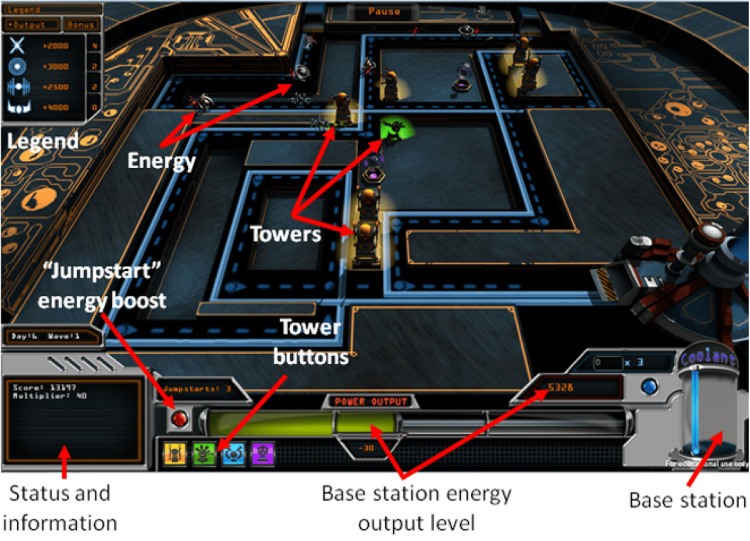
Game-play screenshot

**Figure 3:**
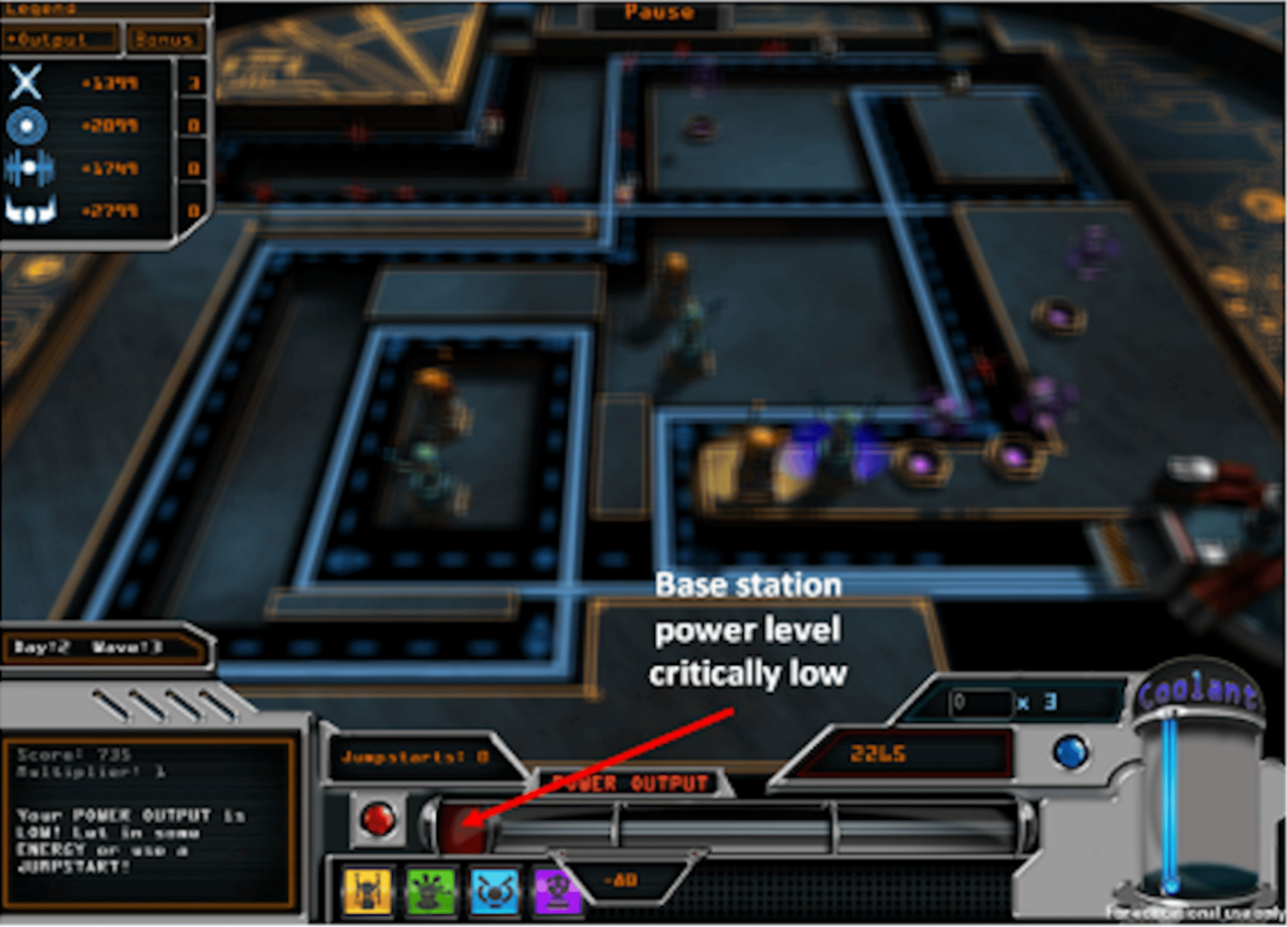
Blurring effect screenshot

**Figure 4:**
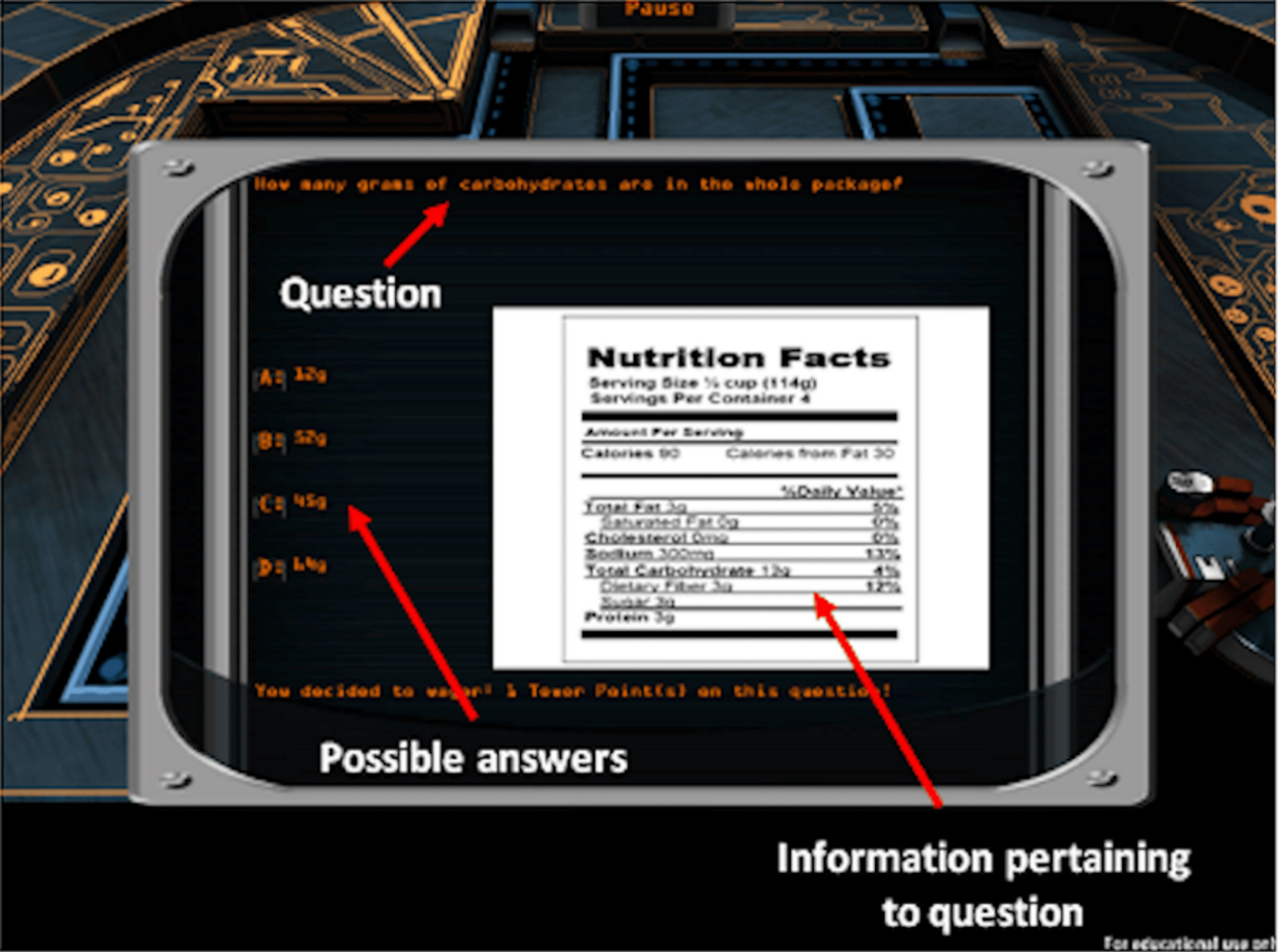
Sample question screenshot

This video game, named “Power Defense”, is based on the tower defense genre of video games (Figures 2-4). Prior to starting the recruitment process for the study, we “alpha” tested the game on eight adolescent volunteers from the diabetes clinic waiting room. Their feedback helped us enhance the graphics, music, and aesthetic appeal of the video game. As well, we used the adolescents’ feedback for addressing technical glitches, such as slow or ineffective transitions between levels.

Intervention: description of the game

Power Defense requires players to balance the energy that goes into a reactor-based power base station (representing a type 1 diabetes patient), which stores energy (representing “blood sugar level”) (Figure 2). Players can place towers, which drain energy (representing exercise), and can use “real-time coolant” (representing short-acting insulin) and “daily super coolant” (representing long-acting insulin) to maintain the desired energy level (representing daily blood sugar levels). Calculations must be performed in real time, which is similar to calculations that diabetes patients have to make for managing their blood sugar levels. During “attack waves," energy entities that represent food try to approach the base station. The player needs to survive as many energy attack waves as possible and keep the base station’s power output at an acceptable level. The challenge is to ensure that an excessive amount of energy does not go through, which would lead to power overload. When the power falls below a pre-set threshold level, the game screen starts to blur (mimicking symptoms of low blood sugar) (Figure 3). When the power output is too high, the player is prompted to inject coolant.

Implicit teaching strategies are employed for the acquisition of diabetes numeracy skills through Power Defense. To support knowledge and skill transfer to diabetes management in real life [17], we also incorporated explicit strategies of teaching; during certain parts of the game, based on user actions, the player is shown diabetes-specific questions, and if they answer correctly, they get rewarded (Figure 4). If their answers are incorrect, they receive the correct answer and specific explanations. The game also tracks various player statistics, including the number of correct or incorrect calculations, the time spent within and outside of the optimal power range, and the number of questions answered correctly.

Conceptual foundation

The video game intervention was informed by experiential learning principles, emphasizing repeated practice with immediate feedback in scenarios that simulate real-world type 1 diabetes decisions [18, 19]. This framing guided both the selection of outcome variables (diabetes numeracy as the proximal target; glycemic control and diabetes-related QL as clinically meaningful downstream outcomes) and the game design features intended to support active, practice-based learning.

Sample

From May 2011 to July 2011, we enrolled 42 study participants from the type 1 diabetes clinic at a tertiary care hospital in Canada. Study participants were 13 to 18 years old, were diagnosed with type 1 diabetes for at least one year (based on Canadian Diabetes Association criteria), and had their initial diabetes education at the hospital diabetes clinic. They were excluded from the study if they had poor proficiency in the English language, had a known learning disability or psychiatric/behavioral diagnosis, or had a visual or hearing impairment. Participants received high school volunteer hours and a total of $45 in iTunes gift cards, given incrementally during the multiple study visits. In addition, their bus fare for transportation to and from the study visits was also provided. This study was approved by the Research Ethics Board of the Hospital for Sick Children, Toronto, Canada (approval number: 1000025846). Regarding the recruitment and consent process, potential participants were identified during routine diabetes clinic visits. A member of the clinical team first introduced the study to patients/caregivers as part of the circle of care; those who expressed interest were then approached by the study investigator to review study details and procedures. Written informed consent was obtained from all participants and from their parent/guardian in attendance, in accordance with ethics approval. Consent was obtained using paper consent forms; patients and caregivers were given time to review the information and ask questions, and were informed that participation was voluntary and could be withdrawn at any time without impact on clinical care.

Recruitment and administration of gaming intervention

Recruitment, administration of the consent process, collection of all data, and administration of the video game intervention were all performed by a single person (author EBa), thereby standardizing the process and minimizing any potential additional confounding variables. EBa recruited potential participants from the waiting room during type 1 diabetes clinics, obtained written consent, and gathered data, including glycemic control (A1C) and demographic information about the participant and one parent in attendance, confirmed by chart review. EBa then administered tests of literacy, general numeracy, diabetes-related numeracy, general and diabetes-specific QL, and diabetes-related problem-solving skills. Demographic and clinical self-reported information included patient and parent age, sex, patient and parent education level, proficiency of math skills at school, video game playing habits, ethnicity, and annual household income. Through examination of the electronic medical records, EBa collected A1C measurements and calculated an average (arithmetic mean) A1C level for the six months prior to enrollment.

The adolescents participated in three video game sessions during which they played the video game for one hour in a designated research room at the study center. They attended three sessions within one week, separated by at least one day between each session. After completion of the third video game session, diabetes numeracy was re-assessed using a modified version of the Adolescent Diabetes Numeracy Test (aDNT14). A month after this session, participants returned to play the game a final time. At this visit, measures of diabetes numeracy, literacy, general numeracy, QL, and problem-solving were reassessed in order to help ascertain if the video game intervention successfully improved diabetes numeracy skills as opposed to improvement in the other domains of general numeracy, literacy, and problem-solving. Similarly, the re-administration of these measures would help explain the reason for the effect on glycemic control, if any. Finally, A1C measurements were again collected through the electronic medical records, and an average (arithmetic mean) A1C level for the six months after completion of the video game intervention was calculated in order to examine the study’s sustained effect on diabetes control.

Instruments

All paper-based questionnaires/tests were administered in person in a private clinic room, and participants were encouraged to take the time they needed to complete the measures. EBa ensured that there were no missing data.

Diabetes-Related Numeracy

Diabetes-specific numeracy builds on general numeracy (e.g., basic arithmetic) but requires applying calculations to diabetes contexts, such as carbohydrate counting, insulin-to-carbohydrate ratios, correction factors, and interpretation of blood glucose values. Diabetes-related numeracy skills were assessed using the adolescent Diabetes Numeracy Test (aDNT-14) [15], which has established validity evidence for use with adolescents with type 1 diabetes. The shortened 14-item version was administered during this study. Each item is scored dichotomously (correct/incorrect), yielding a total score ranging from 0 to 14, with higher scores indicating greater diabetes-specific numeracy. The advantage of the aDNT-14 is that it assesses numeracy skills specifically related to diabetes self-care. The aDNT-14 has limited but acceptable internal consistency and evidence of construct validity through associations with glycemic control and diabetes management behaviors [15]. Participants were provided with a basic function calculator, and no time limit was imposed for completion of the aDNT-14, consistent with the instrument’s intent to assess applied numeracy.

General Numeracy

General numeracy was assessed using the Wide Range Achievement Test, Fourth Edition (WRAT-4), math section. The WRAT-4 is a test that measures general calculation skills [20]. Raw scores are converted to age-normed standard scores, allowing comparison with population norms. Higher scores reflect stronger general mathematical ability. The WRAT-4 math section has strong psychometric properties, including high internal consistency and test-retest reliability [20].

Literacy

Literacy was assessed using the Rapid Estimate of Adolescent Literacy in Medicine (REALM-Teen), a word recognition test used as a screening tool for literacy and a good predictor of general reading ability in English [21]. Participants read aloud a list of medical terms, with one point awarded for each correct word; total scores correspond to grade-level reading categories. Higher scores indicate higher estimated literacy. The REALM-Teen has demonstrated strong internal consistency and criterion validity, showing good correlation with standardized reading measures and clinician-rated literacy [21]. It is commonly used as a brief screening tool rather than a comprehensive assessment of reading comprehension.

Quality of Life

General and diabetes specific QL was assessed using the PedsQL 3.0 inventory. The generic core scale and the diabetes specific module were administered to both parent and child. This tool is used for adolescents with type 1 diabetes as well as their caregiver(s) [22]. Items are rated on a Likert scale and transformed to a 0-100 scale, with higher scores indicating better perceived QL. Scale scores are calculated as the mean of completed items within each domain. The PedsQL 3.0 has strong psychometric properties across pediatric populations, including high internal consistency for total and domain scores, good parent-child agreement, and established construct validity in youth with type 1 diabetes [22]. The instrument is sensitive to disease burden and psychosocial functioning and is widely used in both clinical and research settings.

Glycemic Control (A1C)

Glycemic control was assessed by calculating an arithmetic mean A1C for each patient during the six months prior to enrollment in the study, and six months following completion of the study. An average A1C was chosen in contrast to the most recent single A1C, in order to provide a better reflection of actual patient glycemic control trends. The A1C data were obtained from participants’ medical records.

Statistical analysis

Statistical analysis was performed using R version 4.5.2. (The R Core Team, R Foundation for Statistical Computing, Vienna, Austria). Descriptive statistics, mean and standard deviation for continuous variables, and percentages for categorical variables were calculated to describe the sample. To examine the effect of the video game intervention on A1C, we conducted a repeated-measures ANOVA with the measurement points (pre, immediately post, and delayed post) as the within-subjects factor. To examine the effects of the intervention on aDNT-14, Global QL, and Diabetes QL, we conducted paired samples t-tests on pre- and post scores for each measure. Additionally, regarding the correlations, we used Spearman correlation coefficients, given the modest sample size, to reduce sensitivity to non-normal distributions and outliers. All tests were two-sided with an alpha criterion of .05. In the instance of non-significant results, we conducted subsequent Bayesian analyses using the Bayes Factor R package (Rouder et al.; developed by Morey and Rouder; University of Amsterdam, Netherlands/University of California, Irvine, CA, USA) [23] to investigate the strength of evidence for the null hypothesis. We convert the Bayes factors provided from the package functions BF10, indicating evidence for the alternative hypothesis, to BF01 (1/BF10), representing evidence for the null hypothesis.

## Results

Participant characteristics

From May 2011 to July 2011, 42 patients who met eligibility enrollment criteria were recruited from the type 1 diabetes clinic waiting room at the hospital. Of these patients, 33 (79%) participants completed the study. Of the nine participants who did not complete the study, eight did not present to the first video game session, despite voicing interest and multiple telephone reminders, and one patient did not present to the final video game session due to conflicting time commitments with starting college. Although we did not formally track the total number of patients contacted versus those who accepted or declined to participate, recruitment occurred consecutively during clinic visits, with high uptake among eligible participants (as per anecdotal information from the first author). Characteristics of the 42 participants recruited and the 33 participants who completed the study are presented in Table 1.

**Table 1:** Participant characteristics %: Percentage; n: number; A1C: hemoglobin A1C; aDNT: Adolescent Diabetes Numeracy Test; MDI: multiple daily injection; TID: three times a day; QL: quality of life; REALM-Teen: Rapid Estimate of Adolescent Literacy in Medicine

Characteristic	Patients who completed the study, n=33	Patients who did not complete the study, n=9	P-value
Mean age (years)	16.8	16.9	0.9072
Male (%)	51.50%	55.70%	0.8297
Born in Canada (%)	90.90%	77.80%	0.2808
Mean years of diabetes diagnosis	7.8 years	8.7 years	0.5998
Average A1C at baseline (%)	8.3	8.7	0.6
Average aDNT-14 scores at baseline	10.4	10.1	0.8311
% who completed math level ≤ grade 9 (%)	19/33 (57.6%)	5/9 (55.6%)	0.9136
Average math mark (%)	72.7	78.8	0.2433
% who monitor blood glucose ≤ 2 times/day	3/33 (9.1%)	2/7 (22.2%)	0.2809
% on insulin pump	19/33 (57.6%)	2/9 (22.2%)	0.13
% MDI insulin regimen (≥4 times/day)	8/14 (57.1%)	1/7 (14.3%)	0.1588
% TID insulin regimen (3 times/day)	6/14 (42.9%)	6/7 (85.7%)	0.1588
Average global QL score (0-100)	78.10	76.50	0.73
Average diabetes QL score (0-100)	72.20	71.70	0.93
Average REALM-Teen score (0-66)	61.80	61.10	0.75
% with parental education ≤ high school	7/33 (21.2%)	4/9 (44.4%)	0.2086
% with total household income ≤ $50,000/year	7/33 (21.2%)	3/9 (33.3%)	0.6603

Primary analysis

Diabetes-Related Numeracy and Glycemic Control

**Figure 5:**
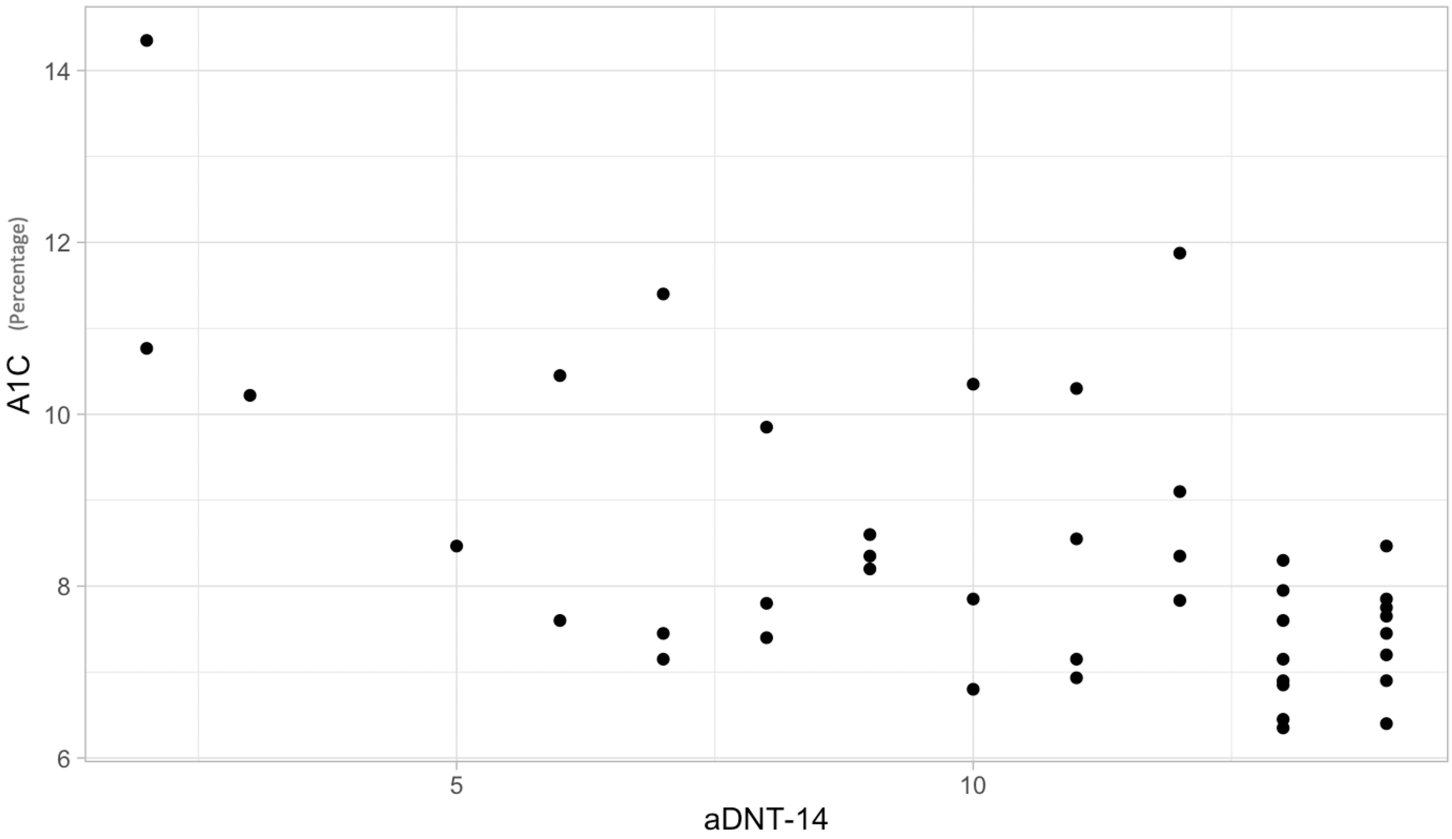
Baseline aDNT-14 and A1C scores aDNT-14: Adolescent Diabetes Numeracy Test; A1C: hemoglobin A1C

We hypothesized that participants with low numeracy test scores at baseline would have higher A1C measurements as compared to participants with higher diabetes numeracy test scores. The Spearman correlations showed a significant negative association between baseline A1C and baseline numeracy test results (Rho = -0.48, p = .001) (Figure 5).

Diabetes-Related Numeracy and QL

We examined the relationship between diabetes-related numeracy and QL measures. Spearman’s rho correlations revealed no significant relationship between aDNT-14 at baseline and Global QL at baseline, Rho = -.03, p = .84. In addition, there was no significant correlation between baseline aDNT-14 and baseline Diabetes QL, Rho = .16, p = .33. Subsequent Bayesian correlational analysis revealed moderate evidence in favor of the null hypothesis for the aDNT-14 and Global QL association (BF01 = 2.8) and anecdotal evidence for the null hypothesis for the association between aDNT-14 and Diabetes QL (BF01 = 2.46).

Intervention Effect on Diabetes-Related Numeracy, A1C, and QL

We conducted a repeated measures ANOVA on aDNT-14 scores across the three time points of measurement: pre (M = 10.3, SD = 3.5), immediately post intervention (M = 10.7, SD = 3.1), and one month following the intervention (M = 10.5, SD = 3.2). There was no significant effect of time on aDNT-14 F(2, 64) = 0.170, p = .84, partial eta squared = .005. A follow-up Bayesian repeated measures ANOVA revealed a Bayes factor for time as BF01 = 10, indicating moderate evidence in favor of the null hypothesis.

A paired samples t-test revealed no significant differences in A1C between pre (M = 8.34, SD = 1.68) and post (M = 8.36 , SD =1.43) intervention t(32) = -1.28, p = .21 Cohen’s D = -.22. 

There was also a non-significant pre (M = 72. 11, SD = 16.47) and post (M = 74. 82 SD =15.40 ) difference for Diabetes QL t(33) = -1.80, p = .08, Cohen’s D = -.31. Finally, we report a non-significant change in Global QL pre (M = 77.80488, SD = 11.97919) and post (M = 79.92188, SD = 14.85963) scores t(33) = -1.07, p =.29 Cohen’s D = -.18. Again, to further investigate these null findings, subsequent Bayesian t-tests were conducted. Results revealed moderate evidence in favor of the null hypothesis for change in A1C (BF01 = 2.5), inconclusive evidence for the null hypothesis for change in Diabetes QL (BF01 = 1.2), and moderate evidence for the null hypothesis for change in Global QL (BF01 = 3.2).

Secondary analysis

We included all 42 participants enrolled to explore associations with baseline characteristics and outcomes. We used Spearman’s correlation coefficient to examine the associations of aDNT-14, REALMteen, WRAT-4, and A1C (Table 2) revealing significant associations across all variables except for A1C and WRAT-4. Subsequent Bayesian correlation for A1C and WRAT-4 revealed inconclusive evidence for the null hypothesis BF01 = .51.

**Table 2:** Spearman's Rho correlation matrix of baseline aDNT-14, REALM-Teen, WRAT-4, and A1C Significance notes; *** < .001,  ** <.01, * <.05 aDNT-14: Adolescent Diabetes Numeracy Test; REALM-Teen: Rapid Estimate of Adolescent Literacy in Medicine; WRAT-4: Wide-Range Achievement Test, Fourth Edition; A1C: hemoglobin A1C

	aDNT-14	REALMteen	WRAT-4	A1C
aDNT-14	1			
REALM-Teen	** 0.56	1		
WRAT-4	*** 0.72	* 0.4	1	
A1C	** -.48	* -0.47	-0.31	1

## Discussion

Our pilot study aimed to first assess the baseline association between numeracy, A1C, and QL of adolescents with type 1 diabetes and second, to explore the impact of a *de novo* video game intervention on these outcome measures. By focusing on adolescents in a Canadian tertiary-care setting, this work extends prior adult-focused literature and addresses a population in which developmental context, caregiver involvement, and technology use may shape how type 1 diabetes-related numeracy relates to outcomes, including glycemic control.

Primary outcomes

This study demonstrates that in adolescents with type 1 diabetes, low numeracy and literacy are statistically significantly associated with worse glycemic control. This association was previously demonstrated modestly in adults with mainly type 2 diabetes and an ethnically relatively homogenous population of adolescents with type 1 diabetes [2, 15]. The participants in our study had diverse social identities (e.g., related to sex, ethnicity/race, etc.), which is highly representative of our urban ethnically diverse clinic population [24].

No statistically significant effect of diabetes numeracy on QL was found in this study. However, QL is complex and multifactorial, and likely not explained by numeracy alone. Life circumstances, such as developmentally appropriate adolescent challenges as well as the burden of a chronic illness, beliefs about illness and treatment, mood, and family dynamics, likely heavily influence QL. Nevertheless, we included QL because it is a patient-centered outcome that captures a multitude of factors that impact the diabetes-related lived experiences of adolescents.

Interestingly, the developmentally appropriate and interactive video game intervention did not lead to a change in diabetes numeracy, A1C, or QL. It is, therefore, likely that the duration of the study and the “dose” (length of time played), as well as the frequency of the video game intervention, were insufficiently short to effect a significant change in these complex and multi-factorial outcome measures. Furthermore, as this is a unique approach to patient education, the application of the video game intervention was quite controlled in terms of location, time played, and frequency. This is contrary to the nature of video games, in which the player engages with the video game at the time and location of his/her choice and plays as frequently as desired. Video games embody experiential learning and, therefore, are best applied as determined by the learner’s need. The controlled application of the video game in our study may have therefore hindered the effects of the video game on numeracy and thereby on A1C and QL. Therefore, these findings support the feasibility and potential value of an experiential, game-based approach for improving type 1 diabetes numeracy while highlighting the need for larger studies with flexible and longer exposure and/or integration into routine education to explore sustained clinical and patient-reported impacts.

Secondary outcomes

The significant association between literacy (REALM-Teen) and numeracy at baseline, as well as literacy and A1C at baseline, is interesting. In our study, participants with high numeracy tended to also score high in literacy, and vice versa. This contrasts with findings from adult studies. Many adult patients have adequate literacy skills but poor numeracy skills. In a study assessing adult patient understanding of food labels, 77% of patients had at least grade 9 literacy skills, but only 37% of these patients had numeracy skills at a grade 9 level [25]. In a cross-sectional study of 3,260 Medicare adult patients with and without diabetes, 23% of patients with adequate literacy skills could not conclude whether a blood glucose value was in the target range [26]. The development of literacy and numeracy skills in children is quite complex and multi-factorial. Literacy and numeracy may be related to parental skills, socioeconomic status, nutritional status, and physical activity levels [27]. In addition, literacy skills develop in a different trajectory than numeracy skills. Literacy seems to develop in a sequential manner, after decoding is mastered, leading to the quantitative acquisition of vocabulary and grammar, as well as improved comprehension. Numeracy, however, tends to involve new conceptual categories, which are built on basic numerical knowledge but are also distinctive categories on their own. Therefore, excellence in one domain (i.e., geometry) does not always predict excellence in a different domain (i.e., calculus) [28]. Research is lacking on how and when numeracy and literacy skills are codependent, and the timing of when this codependence ceases. The finding in our study may indicate that numeracy skill acquisition is still quite dependent on literacy skills, even in the adolescent years. This warrants further investigation as the prevention of poor numeracy skills later in life may require intervention at earlier developmental stages.

Application to practice

Several findings from this study are useful for the multidisciplinary team involved in the care of adolescent patients with type 1 diabetes. Firstly, given the importance of diabetes-specific numeracy and the high-frequency demands of type 1 diabetes self-management (e.g., glycemic control), it is imperative that health care team members are aware of the patient’s numeracy skills before tailoring educational strategies to their needs. Secondly, diabetes numeracy and literacy can and should be assessed at diagnosis to help guide the choice of insulin therapy regimen and before making major changes to the patient’s diabetes regimen, such as initiating more complex insulin pump therapy. Furthermore, the education of adolescents related to numeracy skills needs to be engaging, interactive, and developmentally appropriate, matching the adolescents’ learning preferences and developmental context. An experiential approach, where adolescents repeatedly practice numeracy decisions with feedback in realistic scenarios, may be more engaging and effective than information-only instruction for many adolescents. In addition, secondary findings from the study hint towards the importance of strong literacy skills in association with numeracy. Therefore, literacy, in particular health literacy, may also be worthwhile to assess before tailoring the patient's treatment regimen and educational strategies so that the tailoring is appropriate for their literacy level. Finally, clinicians should maintain awareness of available technology-based education supports, including games and applications, and be prepared to recommend options that align with individual patient needs and available resources. Although most teams would not be able to develop new technology-based resources due to financial and time limitations, identifying and curating high-quality tools could broaden the support offered to patients and caregivers.

Study limitations

There are several limitations to our study. First, as this study explored a novel video game intervention for type 1 diabetes management, it was primarily a pilot study that was quasi-experimental and took place at a single center. Therefore, causal inferences regarding the intervention’s effect are limited, and findings should be interpreted as an initial exploration. Additionally, participant numbers were low for this study, thereby limiting the statistical power that could be applied for our analyses. This is particularly relevant for the instances in which our subsequent Bayes factor analyses yielded anecdotal or inconclusive evidence for the null hypothesis. In these instances, there was a failure to reject the null hypothesis by way of null hypothesis significance testing and low confidence in the null hypothesis itself by way of Bayes factor analysis. This would suggest our study was not powered to adequately detect effects where there was both a non-significant finding and a low Bayes factor for the null hypothesis. In addition, the first part of the study, assessing baseline diabetes numeracy skills in relation to glycemic control and QL, is cross-sectional in nature; therefore, the results reported are informative but can only describe associations. Second, recruitment occurred during routine clinic visits, and we did not officially document response rates; hence, selection bias remains possible. Also, the patients who enrolled in our study were motivated adolescents who generally had good numeracy skills and good A1C measures (mean 8.3) compared with the clinic mean (mean 8.6). Therefore, the video game intervention may not be able to improve their skills further; in essence, this is a ceiling effect. Third, the data were collected in 2011, and diabetes technologies have evolved substantially since then (e.g., wider use of continuous glucose monitoring and more advanced pump systems), which may reduce some numeracy burden in contemporary care. However, diabetes-specific numeracy skills remain necessary because technology does not eliminate decision-making demands and may fail or require manual back-up dosing. Fourth, because it was a pilot study, the location and timing of the video game intervention were limited and controlled, which is against the nature of user-initiated play with video games. This may have, in turn, limited the intervention effect of the video game. Fifth, the questions in the aDNT-14 tool are quite basic and may not sufficiently challenge adolescents who are faced with calculation challenges multiple times per day. It may therefore not be sensitive or specific enough to reflect small changes in numeracy skills. We administered the aDNT-14 at baseline and then re-administered a modified version immediately after the third video game visit and one month after the intervention. The modifications included changing the order of the questions and changing the values within the questions; however, we did not establish validity evidence for the modified version. Sixth, there would be a concern for the testing effect (learning from repeatedly taking the test); however, this was not evident as the aDNT-14 scores were stable with repeated testing. Seventh, this study excluded non-English-speaking patients, who may be immigrants and at higher risk for lower numeracy and worse glycemic control. Eight, A1C is an indirect proxy of overall type 1 diabetes management and is influenced by multiple biological and contextual factors; larger longitudinal studies are needed to clarify pathways linking numeracy, education supports, and clinical outcomes. Finally, in this study, we did not assess parental numeracy skills, which may be important if parents are still involved in their adolescent’s diabetes management. However, we opted not to measure parental numeracy due to feasibility concerns from a time and data collection perspective.

Future directions

A few interesting findings from secondary analyses are worth future exploration. Firstly, further studies are needed to explore the developmental sequence of literacy and numeracy skills in young children, with particular attention to the convergence of the interdependence between these two domains. This will, in turn, help direct numeracy and literacy interventions at the appropriate developmental age group. Finally, it is important to develop a more sensitive tool for assessing numeracy skills. This would enable the health care team to accurately gauge patients’ numeracy skills and capture small but clinically important changes in numeracy.

Specifically, related to primary outcomes in our study, future studies are needed to examine the impact of gaming interventions for patients with low numeracy through a larger study sample and with longer and more flexible exposure to the technology intervention.

## Conclusions

A diabetes focused video game intervention dedicated to literacy and numeracy had no impact on glycemic control. Diabetes related numeracy is a patient characteristic that can and should be assessed at diagnosis and at critical time intervals during insulin management, particularly if one is considering transitioning an adolescent with type 1 diabetes to insulin pump therapy. Assessing diabetes related numeracy and making the team aware of the patient’s numeracy level can help team members tailor their educational strategies. Video games are a developmentally appealing medium for education, as it engages the adolescent in experiential learning. Our study’s limitations, mentioned above, may have impacted the evidence for the success of our video game intervention designed to improve diabetes numeracy. Nonetheless, video games and other interactive technologies should be further studied as numeracy-focused interventions for young patients with diabetes.

## References

[1] Cheng AY (2013). Canadian Diabetes Association 2013 clinical practice guidelines for the prevention and management of diabetes in Canada. Introduction. Can J Diabetes.

[2] Osborn CY, Cavanaugh K, Wallston KA, White RO, Rothman RL (2009). Diabetes numeracy: an overlooked factor in understanding racial disparities in glycemic control. Diabetes Care.

[3] (2003). Building on our competencies: Canadian results of the International Adult Literacy and Skills Survey, 2003 - archived. https://www150.statcan.gc.ca/n1/pub/89-617-x/89-617-x2005001-eng.pdf.

[4] Turrin KB, Trujillo JM (2019). Effects of diabetes numeracy on glycemic control and diabetes self-management behaviors in patients on insulin pump therapy. Diabetes Ther.

[5] Zuijdwijk CS, Cuerden M, Mahmud FH (2013). Social determinants of health on glycemic control in pediatric type 1 diabetes. J Pediatr.

[6] Crain ER, Ramphul R, Butler AM, Huang X, Minard CG, Redondo MJ, DeSalvo DJ (2023). Social determinant of health impact on diabetes device use and clinical outcomes in youth with type 1 diabetes. Pediatr Diabetes.

[7] Markowitz JT, Volkening LK, Laffel LM (2014). Care utilization in a pediatric diabetes clinic: cancellations, parental attendance, and mental health appointments. J Pediatr.

[8] Varni JW, Delamater AM, Hood KK (2018). PedsQL 3.2 diabetes module for children, adolescents, and young adults: reliability and validity in type 1 diabetes. Diabetes Care.

[9] Chan HH, Kwong HY, Shu GL, Ting CY, Lai FH (2021). Effects of experiential learning programmes on adolescent prosocial behaviour, empathy, and subjective well-being: a systematic review and meta-analysis. Front Psychol.

[10] Greenwood DA, Gee PM, Fatkin KJ, Peeples M (2017). A systematic review of reviews evaluating technology-enabled diabetes self-management education and support. J Diabetes Sci Technol.

[11] Swartwout E, El-Zein A, Deyo P, Sweenie R, Streisand R (2016). Use of gaming in self-management of diabetes in teens. Curr Diab Rep.

[12] Thompson CG, von Gillern S (2020). Video-game based instruction for vocabulary acquisition with English language learners: a Bayesian meta-analysis. Educ Res Rev.

[13] Edwards SL, Zarandi A, Cosimini M, Chan TM, Abudukebier M, Stiver ML (2025). Analog serious games for medical education: a scoping review. Acad Med.

[14] Alshammri F, Abdulshakour M, Chen L, Sheppard R, Kearney J, Petropoulos JA, Bilgic E (2025). Pediatric endocrinology education among trainees: a scoping review. Clin Teach.

[15] Mulvaney SA, Lilley JS, Cavanaugh KL, Pittel EJ, Rothman RL (2013). Validation of the diabetes numeracy test with adolescents with type 1 diabetes. J Health Commun.

[16] Mackinnon C, Akhtar-Danesh N, Palombella A, Wainman B (2022). Using Q-methodology to determine students' perceptions of interprofessional anatomy education. Anat Sci Educ.

[17] Sun R, Slusarz P, Terry C (2005). The interaction of the explicit and the implicit in skill learning: a dual-process approach. Psychol Rev.

[18] Falloon G (2019). Using simulations to teach young students science concepts: an experiential learning theoretical analysis. Comput Educ.

[19] Dewi EU, Nursalam Nursalam, Mahmudah Mahmudah, Yunitasari E (2023). The effect of peer support psychoeducation based on experiential learning on self-care demands among breast cancer patients with post-chemotherapy. J Public Health Res.

[20] Hynd G, Reynolds CR, Kamphaus RW (2006). Handbook of psychological and educational assessment of children: intelligence, aptitude, and achievement. Arch Clin Neuropsychol.

[21] Davis TC, Wolf MS, Arnold CL (2006). Development and validation of the Rapid Estimate of Adolescent Literacy in Medicine (REALM-Teen): a tool to screen adolescents for below-grade reading in health care settings. Pediatrics.

[22] Varni JW, Burwinkle TM, Jacobs JR, Gottschalk M, Kaufman F, Jones KL (2003). The PedsQL in type 1 and type 2 diabetes: reliability and validity of the Pediatric Quality of Life Inventory Generic Core Scales and Type 1 Diabetes Module. Diabetes Care.

[23] (2026). Bayesfactor: computation of Bayes factors for common designs. R package version 0.9.12-4.7. https://github.com/richarddmorey/bayesfactor.

[24] Yeshayahu Y, Sochett EB, Deda L, Sud S, Mahmud FH (2012). Type 1 diabetes as a risk factor for impaired vitamin D status in a multi-ethnic cohort of Canadian adolescents. Can J Diabetes.

[25] Rothman RL, Housam R, Weiss H (2006). Patient understanding of food labels: the role of literacy and numeracy. Am J Prev Med.

[26] Gazmararian JA, Baker DW, Williams MV (1999). Health literacy among Medicare enrollees in a managed care organization. JAMA.

[27] Bacon P, Lord RN (2021). The impact of physically active learning during the school day on children's physical activity levels, time on task and learning behaviours and academic outcomes. Health Educ Res.

[28] Purpura DJ, Hume LE, Sims DM, Lonigan CJ (2011). Early literacy and early numeracy: the value of including early literacy skills in the prediction of numeracy development. J Exp Child Psychol.

